# Autistic and transgender/gender diverse people’s experiences of health and healthcare

**DOI:** 10.1186/s13229-024-00634-0

**Published:** 2025-01-21

**Authors:** Kate Green, Elizabeth Weir, Lily Wright, Carrie Allison, Simon Baron-Cohen

**Affiliations:** Department of Psychiatry, Autism Research Centre, University of Cambridge, Douglas House, 18B Trumpington Road, Cambridge, CB2 8AH UK

**Keywords:** Autism, Transgender/gender diverse, Healthcare, Healthcare quality, Physical health, Mental health, Self-harm

## Abstract

**Background:**

Autistic people and transgender/gender diverse people experience poorer healthcare experiences and greater risk of diagnosed, suspected, and assessment recommended health conditions, compared to non-autistic and cisgender individuals, respectively. Despite this, there is a paucity of studies on the healthcare experiences and health outcomes of transgender/gender diverse autistic individuals.

**Methods:**

We compared the healthcare experiences and health outcomes of cisgender autistic (n = 1094), transgender/gender diverse autistic (n = 174), and cisgender non-autistic adults (n = 1295) via an anonymous, self-report survey. All individuals whose sex assigned at birth did not match their current gender identity were categorized as transgender/gender diverse; this was possible to determine, as the survey asked about sex assigned at birth and gender in separate questions. Unfortunately, n = 57 transgender/gender diverse non-autistic participants were excluded from these analyses a priori, due to low power. Unadjusted and adjusted binomial logistic regression models with FDR correction were employed to assess healthcare experiences and rates of co-occurring mental and physical health conditions.

**Results:**

Both transgender/gender diverse and cisgender autistic adults had higher rates of all health conditions (including conditions that are formally diagnosed, suspected, or recommended for assessment), compared to cisgender non-autistic adults. Transgender/gender diverse autistic adults were 2.3 times more likely to report a physical health condition, 10.9 times more likely to report a mental health condition, and 5.8 times more likely to report self-harm than cisgender non-autistic adults. Both autistic groups also reported significantly poorer healthcare experiences across 50/51 items.

**Limitations:**

These data were not originally collected to understand the experiences of transgender/gender diverse individuals. In addition, our recruitment strategies, use of a convenience sampling method, and the use of a self-report survey limit the generalizability of the study. As our sample was biased towards white individuals, UK residents, relatively highly educated individuals, those assigned female at birth, and those who currently identify as female, our findings may be less applicable to individuals of differing demographics. Finally, the present study does not include information on the experiences of transgender/gender diverse non-autistic people.

**Conclusions:**

Autistic people have poorer self-reported health and healthcare; however, being gender diverse is associated with further risk for certain adverse experiences and outcomes. Future research on the health and healthcare experiences of transgender/gender diverse autistic people is urgently needed. In particular, forthcoming studies in this area should aim to recruit large-scale and representative studies and should compare the experiences of transgender/gender diverse autistic people to those of transgender/gender diverse non-autistic people. Greater recognition of challenges and reasonable adjustments are essential for people with marginalized, intersectional identities in clinical practice.

**Supplementary Information:**

The online version contains supplementary material available at 10.1186/s13229-024-00634-0.

## Background

Autism is a heterogenous set of neurodevelopmental conditions characterised by differences in social communication; repetitive and restricted behaviours, interests, or activities; and sensory differences [1]. Recent estimates suggest around 1 in 36 children are autistic, although recognition and diagnosis of autism is increasing over time and estimates vary across studies [2–4]. In this paper we use the term ‘sex’ to refer to an individual’s sex assigned at birth, based on chromosomal, genital, or hormonal characteristics [5]. ‘Gender’ will be used to describe an individual’s identity which may or may not align with their sex assigned at birth [6]. We use the term ‘transgender/gender diverse (TGD)’ to describe the experiences of any individual whose gender identity differs from their sex assigned at birth, including but not limited to individuals who are transgender, non-binary, genderfluid, agender, bigender, gender queer, two-spirit, and others. Individuals whose gender identity aligns with their sex assigned at birth are referred to as ‘cisgender’.

A growing body of literature suggests that TGD individuals may be up to 3.03–7.76 times more likely to be autistic [5, 7–9], and have more autistic traits than others [5, 10]. Additionally, autistic populations are overrepresented at gender clinics, and are more likely to report gender diversity and gender-dysphoric traits than non-autistic people [11, 12].

Autistic people experience far higher rates of physical and mental health conditions than others, including chronic and life-threatening conditions [13–20]. Regarding physical conditions, higher rates have been found across all organ systems suggesting an increased overall health burden [20]. Examples of conditions include cardiovascular disease, epilepsy, sleep disorders, gastrointestinal disorders, neurological conditions, immune conditions, endocrine conditions, reproductive health conditions, attention deficit hyperactivity disorder (ADHD), anxiety, sleep–wake disorders, depressive disorders, OCD, bipolar, and schizophrenia [21–25]. Compared to non-autistic individuals, autistic individuals without intellectual disability have a 1.71 times greater mortality rate [26], and as many as 1 in 4 autistic adults experience incomplete suicide [27, 28]. While the literature is comparatively sparse for TGD populations, studies have found an increased likelihood of diabetes, cardiovascular diseases, arthritis, dementia, and mental health conditions such as depression and anxiety compared to cisgender individuals [29–32]. Further, TGD individuals have elevated overall mortality compared with cisgender individuals [33], and 32–50% of TGD individuals report experiences of incomplete suicide [34].

Both autistic and TGD people report practitioner and system level barriers to adequate healthcare. Autistic individuals report difficulties navigating the healthcare system, including inflexible appointment structures and limited availability of formal or informal support [20, 35]. Healthcare professionals report limited knowledge, resources, and training regarding autistic patients [36], may use inaccessible language, and may be unwilling to make accommodations [35]. Barriers specific to autistic individuals’ needs have also been identified, such as diagnostic overshadowing [25] and difficulties with communication, sensory sensitivities, bodily awareness, and information processing within healthcare [19, 35, 37, 38]. System-level barriers for TGD individuals include the absence of transgender-focused medical curricula and training, shortages of specialist centres (and thus long waiting times), legislative restrictions on gender-affirming healthcare especially for transgender youth, and technical barriers regarding records of gender and name [39–42]. Practitioner level barriers include reports of transphobia and even refusal of care due to gender identity [39, 43]. Compared to cisgender individuals, TGD people also have relatively higher levels of unmet healthcare needs, lower satisfaction with their healthcare, less positive interpersonal communication with practitioners, and poorer overall primary care experiences [32, 44, 45].

Despite findings that TGD people are more likely to be autistic, that both groups have a greater health burden, and that both groups experience poorer quality healthcare, few studies have considered how the intersection of these identities relates to health. In terms of health conditions, LGBTQ+ autistic individuals report a greater number of days of ‘poor’ mental and physical health per month than heterosexual, cisgender autistic individuals [46], although this is yet to be tested in TGD autistic samples alone (since this study was based on a convenience sample of 19 individuals and lacked data on the specific LGBTQ+ identities of the participants). Further, higher rates of mental health needs, difficulties, and psychiatric diagnoses have been found in TGD autistic individuals compared to their cisgender non-autistic counterparts [12, 47, 48]. Among transgender youth, those who are also autistic are more likely to have engaged in self-harm and reckless behaviour to purposely put their life at risk; they are also more likely to have experienced suicidal thoughts and incomplete suicide [48]. In terms of healthcare experiences, qualitative research has identified barriers, such as having gender identity undermined by professionals who wrongly assume that autistic patients are unable to fully understand and narrate their gender identity [40, 43]. Studies also highlight difficulties autistic individuals face with disclosing gender identity to providers, which may relate to healthcare professionals lacking knowledge about either identity, let alone their intersection [49, 50].

There is a lack of large-scale well-powered studies examining how gender identity and autism may interact to influence health and healthcare experiences in adult populations. Further, no studies have yet attempted to quantify any such disparities in both mental and physical health, as well as healthcare experiences.

Intersectionality theory posits the effect of having multiple oppressed social identities may be greater than the sum of each of them [51]. This framework has been applied to explore both LGBTQ+ and disabled identities, and how their coexistence may result in multidimensional, nuanced experiences [52, 53]. For example, there may be a cumulative discriminatory impact of being both autistic and TGD which is qualitatively different from the discrimination associated with each identity separately [54]. Adopting an intersectionality framework, the current study aims to examine whether autistic and TGD identities are associated with poorer health-related outcomes by comparing both the health outcomes and healthcare experiences of cisgender non-autistic, cisgender autistic and TGD autistic adults.

## Methods

### Procedure

The current analyses use data collected for a larger study at the Autism Research Centre (ARC) at the University of Cambridge which compared the prevalence of chronic health conditions and the quality of healthcare between autistic and non-autistic adults [19]. An anonymised self-report survey was administered online using Qualtrics survey software. Information from the National Health Service (NHS), National Institute for Health and Care Excellence (NICE), and the World Health Organization (WHO) was used to develop survey items. All data were collected between July 2019 and January 2021.

### Participants

A cross-sectional, convenience sampling design was used to recruit participants. The study was advertised through social media (Twitter, Facebook and Reddit), the Cambridge Autism Research Database (CARD), Autistica’s Discover Network, and other autism-related organisations and charities, allowing the recruitment of an international and diverse cohort. Participants were not paid, and the only eligibility criteria was that participants were at least 16 years of age and that they gave informed consent. Since the study was advertised through autism-related organisations and described as aiming to understand differences/difficulties autistic people may face when receiving healthcare, our non-autistic group may have been biased towards those interested in autism, for example those who suspected they might be autistic. Whilst advertisement to the general population through social media was used in attempts to mitigate this bias, we additionally excluded the following participants from all groups: those who reported suspected autism, a self-diagnosis, or who were awaiting an autism assessment. These individuals were also excluded since they would not be expected to receive reasonable adjustments relating to autism in their healthcare. Due to the study description, the sample may also be biased towards people with health conditions or who have particularly strong feelings about their healthcare experiences.

Of the individuals who accessed the survey (N = 4158), 33.6% (N = 1396) were excluded due to failure to consent, incomplete demographic information, or unconfirmed age. Individuals with unconfirmed autism status (N = 26) were also excluded. Since all participants were anonymous, an algorithm was used to exclude duplicate responses (N = 112) where participant records matched across 12 criteria (autism diagnosis (yes/no), specific autism diagnosis, type of diagnosing practitioner, year of autism diagnosis, autistic family members (yes/no), age, country of residence, sex assigned at birth, current gender identity, education level, ethnicity, and AQ-10 score). TGD non-autistic respondents (n = 57) were also excluded after performing a priori power calculations, due to small sample size. Finally, as our sample was heavily biased towards individuals whose sex assigned at birth was female, it was essential that sex assigned at birth was included as a covariate in our statistical modelling. Thus, due to perfect separation issues associated with this covariate, four intersex individuals were excluded from the sample. The final sample consisted of n = 2563 participants (n = 1295 cisgender non-autistic, n = 1094 cisgender autistic, n = 174 TGD autistic).

### Measures

#### Demographic information

Demographic information collected included age, sex assigned at birth (male, female or other), current gender identity (male, female, non-binary or other), ethnicity, country of residence, autistic familial relatives (yes or no), highest level of qualification (as a proxy for socio-economic status), employment status, and the AQ-10 [55], a brief measure of autistic traits. Participants were categorised as cisgender if they reported both their sex assigned at birth and current gender identity as ‘male’, or both as ‘female’. All other participants were categorised as TGD, including those who disclosed a ‘male’ sex and ‘female’ gender identity, a ‘female’ sex and ‘male’ gender identity, or either sex and a ‘non-binary’ or ‘other’ gender identity.

#### Healthcare experiences

Healthcare experiences were assessed in the present study using 56 survey items across the domains of (1) general healthcare experiences, (2) communication, (3) anxiety, (4) access and advocacy, (5) system-level problems, (6) sensory experiences, (7) shutdowns, (8) meltdowns, and (9) autism specific experiences. Only participants who reported an autism diagnosis responded to the five questions regarding autism specific experiences. Participants responded regarding their experiences with ‘healthcare professionals’, which were defined to include Doctors, General Practitioners, Nurse Practitioners, Nurses, and Physician’s Assistants. Questions were multiple choice or made on a 4-point Likert Scale, with options being ‘Definitely Agree’, ‘Slightly Agree’, ‘Slightly Disagree’, and ‘Definitely Disagree’. Further information on the content of each survey section can be found in Tables 2 and 3.

#### Health outcomes

Health outcomes across the physical and mental health domains were assessed using survey items which asked participants to disclose whether they had ever received a diagnosis, suspected the condition, or whether a healthcare provider had recommended an assessment, for each condition. Further information on the contents of each section of the survey and the health conditions assessed can be found in Tables 4 and 5.

### Data analysis

Analysis was conducted using R version 4.2.2. Descriptive statistics were calculated for the demographic information using the ‘CrossTable’ function of the ‘gmodels’ package, including factorial ANOVAs (for age and AQ-10 score) and Chi-Square tests (for all other demographic variables). To improve interpretability of results, responses regarding healthcare experiences provided via a Likert-scale were simplified into the binary form of ‘agree’ or ‘disagree’.

Unadjusted and adjusted binomial logistic regression models were conducted to identify group differences in healthcare experiences and in health conditions between (i) cisgender autistic, TGD autistic, and cisgender non-autistic adults, as well as directly between (ii) cisgender autistic and TGD autistic adults. Specifically, the present study compared rates of diagnosed, suspected, and assessment recommendations for mental health conditions and physical health conditions overall. Models were also run to establish differences in rates of each specific diagnosed mental and physical health condition (except for dementia and strokes due to low response rate). Models assessed group differences across each of the 56 survey items under the domains of general healthcare experiences, communication, anxiety, access and advocacy, system-level problems, sensory experiences, shutdowns, and meltdowns. Additional models were conducted to compare autism specific experiences between cisgender autistic and TGD autistic groups only. The Benjamini–Hochberg procedure was used to adjust all obtained *p*-values and control the False Discovery Rate (FDR) [56]. Controlling the FDR in the context of multiple hypothesis testing reduces the likelihood of type II errors [57]. A significance threshold of *p* < 0.05 was used for all analyses, including the final FDR-adjusted analyses.

Adjusted analyses included the covariates of age, sex assigned at birth, ethnicity, country of residence, and education level. Sex assigned at birth was coded as ‘male’ or ‘female’. Low response rates from non-white ethnicities meant that a binary representation of ethnicity (‘white’ versus ‘non-white’) was used. Due to low response rates from non-UK and non-US residents, participants from all other countries of residence were coded as ‘other’. Education level was coded as a categorical variable with the options for highest qualification held being ‘No formal qualifications’, ‘Secondary School/High School level qualifications’, ‘Further vocational qualifications’, ‘University Undergraduate level qualifications’, and ‘University Postgraduate level qualifications’.

While all survey items related to demographics and health outcomes were compulsory, questions regarding healthcare experiences were optional. As a result, a small number of participants did not respond to each individual question relating to healthcare experiences. Further information regarding the sample sizes/missing data for each outcome can be found in the supplementary file in Table S2 for the comparison of cisgender autistic and TGD autistic adults and cisgender non-autistic adults and Table S3 for the comparison between cisgender autistic and TGD autistic adults.

### Community engagement

Our community engagement for the project involved activities both during the development of the project and after data analysis for this study. Before the study began, feedback was provided by two autistic adults via in-person interviews in order to revise and finalise survey questions. In addition, in order to gather insights on the specific experiences of autistic TGD people, results were discussed in an online focus group comprising of ten autistic adults, most of whom were also TGD. This focus group helped to inform our interpretation of the findings, particularly by highlighting areas that were not explored within the context of this study, but which should be considered for future research. Email invitations were sent to members of the Cambridge Autism Research Database (CARD) for both community engagement activities, and individuals could participate via video, audio, or chat during our focus group to account for all communication styles/preferences, and to allow participants to remain anonymous to other participants if desired.

## Results

The majority of the sample were white (82%), UK residents (55%), assigned female at birth (63%), currently identified as female (58%), and had a university education (66%). These biases were also present in each of the three groups separately (except for the TGD group, whose most common gender identity was ‘other’). The mean ages were 38.9 years (SD = 16.1) for the cisgender autistic group, 35.6 years (SD = 13.9) for the TGD autistic group, and 42.1 years (SD = 14.4) for the cisgender non-autistic group. There were significant differences across all demographic characteristics between the three groups, as well as between the autistic groups (except for ethnicity, which was not significantly different between the TGD autistic vs cisgender autistic adults). A full summary of demographic information can be found in Table 1 and a summary of demographic information for autistic participants only can be found in Table S1 of the supplementary file.

**Table 1:** Participant demographics

Characteristics	Cisgender autistic (n = 1094)	TGD autistic (n = 174)	Cisgender non-autistic (n = 1295)	p-values (Sig.)
Age (years), mean (SD)	42.12 (14.40)	35.59 (13.91)	38.88 (16.11)	4.37 × ${10}^{-7}$ (***)
*Age (years), categories, N (%)*				
16–29	262 (23.95)	73 (41.95)	454 (35.06)	
30–39	226 (20.66)	41 (23.56)	260 (20.08)	
40–49	240 (21.94)	28 (16.09)	229 (17.68)	
50–59	229 (20.93)	22 (12.64)	190 (14.67)	
60–69	106 (9.69)	5 (2.87)	106 (8.19)	
70+	31 (2.83)	5 (2.87)	56 (4.32)	
Biological sex, N (%)				3.26 × ${10}^{-7}$ (***)
Female	665 (60.79)	143 (82.18)	811 (62.63)	
Male	429 (39.21)	31 (17.82)	484 (37.38)	
Current gender identity, N (%)				
Female	665 (60.79)	8 (4.60)	811 (62.63)	
Male	429 (39.21)	26 (14.94)	484 (37.38)	
Non-Binary	0 (0)	20 (11.49)	0 (0)	
Other	0 (0)	120 (68.97)	0 (0)	
Ethnicity, N (%)				2.27 × ${10}^{-14}$ (***)
White	950 (86.84)	142 (81.61)	1013 (78.22)	
Non-white	144 (13.16)	32 (18.39)	282 (21.78)	
African	5 (0.46)	0 (0)	12 (0.93)	
Arab	1 (0.09)	0 (0)	8 (0.62)	
Asian	9 (0.82)	0 (0)	42 (3.24)	
Bangladeshi, Indian, Pakistani	8 (0.73)	2 (1.15)	52 (4.02)	
Caribbean	6 (0.55)	1 (0.58)	1 (0.08)	
Hispanic	11 (1.01)	0 (0)	32 (2.47)	
Jewish	24 (2.19)	1 (0.58)	35 (2.70)	
Turkish	1 (0.09)	0 (0)	10 (0.77)	
Mixed race	53 (4.85)	23 (13.22)	65 (5.02)	
Other	26 (2.38)	5 (2.87)	25 (1.93)	
Country of residence, N (%)				2.60 × ${10}^{-36}$ (***)
UK	740 (67.64)	90 (51.72)	574 (44.32)	
USA	107 (9.78)	34 (19.54)	138 (10.66)	
Other	247 (22.58)	50 (28.74)	583 (45.02)	
Australia	14 (1.28)	6 (3.45)	34 (2.63)	
Canada	32 (2.93)	8 (4.60)	50 (3.86)	
Germany	29 (2.65)	10 (5.75)	27 (2.09)	
Netherlands	21 (1.92)	4 (2.30)	33 (2.55)	
Other	151 (13.80)	22 (12.64)	439 (33.90)	
Education, N (%)				1.40 × ${10}^{-7}$ (***)
No formal education	47(4.30)	10 (5.75)	23 (1.78)	
Secondary School/High School	173 (15.81)	43 (24.71)	230 (17.76)	
Further vocational qualifications	176 (16.09)	24 (13.79)	145 (11.20)	
University undergraduate	352 (32.18)	43 (24.71)	390 (30.12)	
University postgraduate	346 (31.63)	54 (31.03)	507 (39.15)	
AQ-10 Score, mean (SD)	7.93 (1.90)	8.51 (1.66)	3.66 (2.55)	< 2 × ${10}^{-16}$ (***)

*SD* standard deviation

*p*-values from Pearson’s Chi-Square test (for categorical variables) or Mann–Whitney U test (means for continuous variables)

Sig. = significance level

*p*-value: < .0.05 = *; < .0.01 = **; < .0.001 = ***

### Healthcare experiences

TGD autistic and cisgender autistic adults both reported significantly poorer healthcare experiences than cisgender non-autistic adults across 50/51 items across the areas of general healthcare experiences, communication, anxiety, access and advocacy, system-level problems, sensory experiences, triggers for a shutdown, and triggers for a meltdown. Compared to cisgender non-autistic individuals, TGD autistic individuals had greater likelihood of healthcare coverage/insurance, (adjusted odds ratio (AOR): 2.30; 95% CI 1.34, 4.17) whereas no significant difference for this item was found between cisgender autistic and cisgender non-autistic individuals. However, both autistic groups reported poorer healthcare experiences for all other items compared to cisgender non-autistic adults. Compared to cisgender non-autistic adults, cisgender autistic adults were 3–6 times more likely and TGD autistic adults were 3–11 times more likely to endorse items regarding anxiety around a common healthcare-related scenario. Further, compared to cisgender non-autistic adults, cisgender autistic adults were 4–8 times more likely and TGD autistic adults were 5–10 times more likely to report a shutdown or meltdown due to a common healthcare-related scenario. For every ten cisgender non-autistic adults, only two cisgender autistic adults and one TGD autistic adult reported (i) understanding what their healthcare professional means when discussing their health (cisgender autistic: AOR = 0.20, 95% CI, 0.15, 0.27; TGD autistic: AOR = 0.14, 95% CI 0.09, 0.21), (ii) knowing what is expected of them when going to see a healthcare professional (cisgender autistic: AOR = 0.23, 95% CI 0.18, 0.28; TGD autistic: AOR = 0.13, 95% CI, 0.09, 0.18), and (iii) being able to describe how bad their pain feels (cisgender autistic: AOR = 0.19, 95% CI 0.15, 0.23; TGD autistic: AOR = 0.13, 95% CI 0.09, 0.18).

When directly comparing healthcare experiences between cisgender autistic and TGD autistic adults, TGD autistic adults reported significantly poorer healthcare experiences across 5/56 measures. These disparities related to communication, access and advocacy, and shutdowns. However, TGD autistic adults were over twice as likely to have health insurance compared to cisgender autistic adults (OR = 2.31, 95% CI 1.35, 4.20). Full results can be found in Tables 2 and 3.

**Table 2:** Self-reported healthcare experiences for cisgender autistic adults and transgender autistic adults compared to cisgender non-autistic adults

	Cisgender autistic adults				Transgender autistic adults			
	Unadjusted		Adjusted model^a^		Unadjusted		Adjusted model^a^	
	OR (95% CI)	*p-*value	OR (95% CI)	*p-*value	OR (95% CI)	*p-*value	OR (95% CI)	*p-*value
*General healthcare experiences*								
Are you able to see healthcare professionals as often as you would like?	0.35 (0.29, 0.42)	4.00 × ${10}^{-16}$ (***)	0.40 (0.33, 0.48)	4.67 × ${10}^{-16}$ (***)	0.26 (0.19, 0.35)	4.00 × ${10}^{-16}$ (***)	0.29 (0.21, 0.40)	4.49 × ${10}^{-13}$ (***)
Do you have health insurance?	0.70 (0.57, 0.85)	5.73 × ${10}^{-4}$ (***)	0.97 (0.78, 1.22)	0.83	1.84 (1.12, 3.21)	0.02 (*)	2.30 (1.34, 4.17)	5.00 × ${10}^{-3}$ (**)
*Communication*								
I am usually able to explain what my symptoms are	0.19 (0.15, 0.24)	4.00 × ${10}^{-16}$ (***)	0.19 (0.15, 0.25)	4.67 × ${10}^{-16}$ (***)	0.13 (0.09, 0.18)	4.00 × ${10}^{-16}$ (***)	0.16 (0.11, 0.23)	4.67 × ${10}^{-16}$ (***)
I usually understand what my healthcare professional means when they discuss my health	0.18 (0.13, 0.24)	4.00 × ${10}^{-16}$ (***)	0.20 (0.15, 0.27)	4.67 × ${10}^{-16}$ (***)	0.11 (0.07, 0.17)	4.00 × ${10}^{-16}$(***)	0.14 (0.09, 0.21)	4.67 × ${10}^{-16}$ (***)
I do not usually ask all the questions I would like to about my health	2.79 (2.33, 3.34)	4.00 × ${10}^{-16}$ (***)	2.62 (2.17, 3.18)	4.67 × ${10}^{-16}$ (***)	3.02 (2.08, 4.49)	2.15 × ${10}^{-8}$ (***)	2.45 (1.67, 3.68)	9.34 × ${10}^{-6}$ (***)
I can bring up a health concern even if my healthcare professional doesn’t ask about it	0.38 (0.32, 0.46)	4.00 × ${10}^{-16}$ (***)	0.37 (0.31, 0.45)	4.67 × ${10}^{-16}$ (***)	0.24 (0.17, 0.34)	4.00 × ${10}^{-16}$ (***)	0.29 (0.21, 0.41)	2.34 × ${10}^{-12}$ (***)
I know what is expected of me when I go to see my healthcare professional	0.23 (0.19, 0.28)	4.00 × ${10}^{-16}$ (***)	0.23 (0.18, 0.28)	4.67 × ${10}^{-16}$ (***)	0.11 (0.08, 0.16)	4.00 × ${10}^{-16}$ (***)	0.13 (0.09, 0.18)	4.67 × ${10}^{-16}$ (***)
*Anxiety*								
The idea of going to see a healthcare professional makes me feel anxious	2.98 (2.46, 3.63)	4.00 × ${10}^{-16}$ (***)	2.94 (2.40, 3.62)	4.67 × ${10}^{-16}$ (***)	4.87 (3.09, 8.10)	1.50 × ${10}^{-10}$ (***)	8.83 (2.41, 6.42)	9.67 × ${10}^{-8}$ (***)
The environment of the waiting room office makes me feel anxious	4.94 (4.11, 5.96)	4.00 × ${10}^{-16}$ (***)	5.02 (4.12, 6.13)	4.67 × ${10}^{-16}$ (***)	4.84 (3.32, 7.23)	3.25 × ${10}^{-15}$ (***)	3.84 (2.61, 5.78)	5.09 × ${10}^{-11}$ (***)
I feel anxious when I see a different healthcare professional to whom I expect	5.93 (4.90, 7.20)	4.00 × ${10}^{-16}$ (***)	6.42 (5.23, 7.92)	4.67 × ${10}^{-16}$ (***)	13.09 (7.88, 23.44)	4.00 × ${10}^{-16}$ (***)	11.30 (6.73, 20.40)	4.67 × ${10}^{-16}$ (***)
The process of setting up an appointment makes me anxious	4.24 (3.51, 5.16)	4.00 × ${10}^{-16}$ (***)	4.67 (3.80, 5.75)	4.67 × ${10}^{-16}$ (***)	9.33 (5.61, 16.70)	1.21 × ${10}^{-15}$ (***)	7.84 (4.68, 14.13)	3.51 × ${10}^{-13}$ (***)
The process of picking up a prescription makes me anxious	4.22 (3.55, 5.05)	4.00 × ${10}^{-16}$ (***)	4.21 (3.48, 5.10)	4.67 × ${10}^{-16}$ (***)	6.22 (4.43, 8.80)	4.00 × ${10}^{-16}$ (***)	5.12 (3.61, 7.31)	4.67 × ${10}^{-16}$ (***)
I frequently leave my healthcare professional’s office feeling as though I did not receive any help at all	3.71 (2.13, 4.41)	4.00 × ${10}^{-16}$ (***)	3.54 (2.96, 4.24)	4.67 × ${10}^{-16}$ (***)	3.49 (2.51, 4.87)	2.33 × ${10}^{-13}$ (***)	2.92 (2.09, 4.10)	7.56 × ${10}^{-10}$ (***)
*Access and advocacy*								
I know who to contact if I have a healthcare concern	0.49 (0.39, 0.61)	2.81 × ${10}^{-10}$ (***)	0.47 (0.37, 0.59)	5.40 × ${10}^{-10}$ (***)	0.31 (0.22, 0.45)	4.93 × ${10}^{-10}$ (***)	0.35 (0.24, 0.51)	6.72 × ${10}^{-8}$ (***)
If I need to go to see a healthcare professional, I am able to get there	0.31 (0.23, 0.41)	2.63 × ${10}^{-15}$ (***)	0.34 (0.25, 0.46)	9.11 × ${10}^{-12}$ (***)	0.12 (0.08, 0.19)	4.00 × ${10}^{-16}$ (***)	0.15 (0.10, 0.23)	4.67 × ${10}^{-16}$ (***)
I usually bring someone along to help support me in my appointments	1.99 (1.65, 2.41)	1.86 × ${10}^{-12}$ (***)	2.29 (1.85, 2.85)	6.64 × ${10}^{-14}$ (***)	2.87 (2.05, 4.00)	9.79 × ${10}^{-10}$ (***)	2.40 (1.67, 3.44)	2.63 × ${10}^{-6}$ (***)
If I need to go to the pharmacy, I am able to get there	0.27 (0.18, 0.39)	1.02 × ${10}^{-11}$ (***)	0.35 (0.23, 0.51)	1.38 × ${10}^{-7}$ (***)	0.13 (0.08, 0.21)	4.00 × ${10}^{-16}$ (***)	0.16 (0.10, 0.26)	1.62 × ${10}^{-12}$ (***)
I am able to follow a procedure for next steps if asked (for example, I will attend follow-up appointments, annual checkups if applicable, etc.)	0.36 (0.27, 0.47)	6.65 × ${10}^{-14}$ (***)	0.35 (0.26, 0.46)	6.13 × ${10}^{-13}$ (***)	0.30 (0.14, 0.30)	5.48 × ${10}^{-15}$ (***)	0.23 (0.15, 0.35)	3.12 × ${10}^{-12}$ (***)
I am able to make appointments for myself	0.36 (0.27, 0.47)	2.51 × ${10}^{-13}$ (***)	0.32 (0.23, 0.43)	3.51 × ${10}^{-13}$ (***)	0.22 (0.15, 0.33)	5.38 × ${10}^{-13}$ (***)	0.25 (0.16, 0.39)	1.29 × ${10}^{-9}$ (***)
I will wait until it is an emergency before I go to see a healthcare professional	1.72 (1.46, 2.04)	3.37 × $${10}^{-10}$$ (***)	1.61 (1.35, 1.92)	1.41 × $${10}^{-7}(***)$$	2.21 (1.57, 3.14)	8.21 × $${10}^{-6}$$ (***)	1.98 (1.40, 2.83)	1.52 × $${10}^{-4}$$ (***)
Chosen not to go in to see a healthcare professional regarding a health concern	1.94 (1.61, 2.33)	5.78 × ${10}^{-12}$ (***)	2.14 (1.75, 2.61)	1.31 × ${10}^{-13}$ (***)	4.37 (2.74, 7.36)	5.35 × ${10}^{-9}$ (***)	3.88 (2.41, 6.59)	1.50 × ${10}^{-7}$ (***)
*System*								
In most appointments, I have enough time to discuss my concerns with healthcare professionals	0.30 (0.25, 0.36)	4.00 × ${10}^{-16}$ (***)	0.33 (0.27, 0.39)	4.67 × ${10}^{-16}$ (***)	0.26 (0.19, 0.37)	1.08 × ${10}^{-14}$ (***)	0.29 (0.21, 0.41)	2.63 × ${10}^{-12}$ (***)
If I need to go to see a specialist for a healthcare concern, I am able to do so	0.40 (0.33, 0.48)	4.00 × ${10}^{-16}$ (***)	0.47 (0.38, 0.57)	2.30 × ${10}^{-13}$ (***)	0.23 (0.17, 0.33)	4.00 × ${10}^{-16}$ (***)	0.27 (0.19, 0.39)	4.82 × ${10}^{-13}$ (***)
I often choose not to go to the doctor with concerns if I need to see a specialist because I know that it will take me many appointments before I can see the specialist	2.02 (1.70, 2.39)	6.39 × ${10}^{-16}$ (***)	1.94 (1.63, 2.32)	4.49 × ${10}^{-13}$ (***)	2.97 (2.13, 4.19)	3.49 × ${10}^{-10}$ (***)	2.53 (1.80, 3.58)	1.79 × ${10}^{-7}$ (***)
I usually leave my appointments knowing what the next steps are (i.e. follow-up appointments, medications, etc)	0.39 (0.32, 0.48)	4.00 × ${10}^{-16}$ (***)	0.42 (0.33, 0.52)	3.67 × ${10}^{-15}$ (***)	0.30 (0.21, 0.43)	2.76 × ${10}^{-11}$ (***)	0.37 (0.26, 0.53)	6.94 × ${10}^{-8}$ (***)
I am provided with appropriate support after I receive a diagnosis of any kind (i.e. anything from infections to chronic conditions)	0.22 (0.18, 0.26)	4.00 × ${10}^{-16}$ (***)	0.24 (0.20, 0.29)	4.67 × ${10}^{-16}$ (***)	0.17 (0.12, 0.24)	4.00 × ${10}^{-16}$ (***)	0.20 (0.14, 0.28)	4.67 × ${10}^{-16}$ (***)
*Sensory experiences*								
Reported at least one sensory difference (hyper- or hyposensitivity)	16.25 (12.72, 21.00)	4.00 × ${10}^{-16}$ (***)	18.61 (14.36, 24.43)	4.67 × ${10}^{-16}$ (***)	55.96 (23.53, 182.53)	4.89 × ${10}^{-15}$ (***)	53.26 (22.27, 174.24)	1.48 × ${10}^{-14}$ (***)
I am able to describe how my symptoms feel in my body	0.17 (0.14, 0.21)	4.00 × ${10}^{-16}$ (***)	0.17 (0.14, 0.21)	4.67 × ${10}^{-16}$ (***)	0.13 (0.09, 0.18)	4.00 × ${10}^{-16}$ (***)	0.16 (0.11, 0.23)	4.67 × ${10}^{-16}$ (***)
I am able to describe how bad my pain feels	0.20 (0.16, 0.24)	4.00 × ${10}^{-16}$ (***)	0.19 (0.15, 0.23)	4.67 × ${10}^{-16}$ (***)	0.11 (0.07, 0.15)	4.00 × ${10}^{-16}$ (***)	0.13 (0.09, 0.18)	4.67 × ${10}^{-16}$ (***)
I am able to describe my sensory processing differences to healthcare professionals	0.47 (0.38, 0.58)	5.64 × $${10}^{-12}$$ (***)	0.45 (0.36, 0.57)	1.59 × $${10}^{-11}$$ (***)	0.37 (0.26, 0.53)	1.02 × $${10}^{-7} (***)$$	0.44 (0.31, 0.64)	1.84 × $${10}^{-5}$$ (***)
The sensory environment of the waiting room is more overwhelming than other environments	5.42 (4.54, 6.47)	4.00 × ${10}^{-16}$ (***)	5.63 (4.67, 6.81)	4.67 × ${10}^{-16}$ (***)	5.62 (3.98, 8.03)	4.00 × ${10}^{-16}$ (***)	4.80 (3.38, 6.91)	4.67 × ${10}^{-16}$ (***)
The sensory environment of the office is more overwhelming than other environments	4.39 (3.68, 5.23)	4.00 × ${10}^{-16}$ (***)	4.40 (3.66, 5.30)	4.67 × ${10}^{-16}$ (***)	3.85 (2.78, 5.34)	1.17 × ${10}^{-14}$ (***)	3.41 (2.44, 4.76)	1.04 × ${10}^{-12}$ (***)
My senses frequently overwhelm me so that I have trouble focusing on conversations with healthcare professionals	8.14 (6.73, 9.89)	4.00 × ${10}^{-16}$ (***)	7.95 (6.50, 9.76)	4.67 × ${10}^{-16}$ (***)	12.27 (8.60, 17.74)	4.00 × ${10}^{-16}$ (***)	10.27 (7.13, 14.98)	4.67 × ${10}^{-16}$ (***)
*Triggers for a shutdown*								
The idea of going to see a healthcare professional	5.61 (4.49, 7.04)	4.00 × ${10}^{-16}$ (***)	5.89 (4.66, 7.50)	4.67 × ${10}^{-16}$ (***)	8.95 (6.26, 12.84)	4.00 × ${10}^{-16}$ (***)	7.79 (5.40, 11.28)	4.67 × ${10}^{-16}$ (***)
Setting up an appointment to see a healthcare professional	5.20 (4.14, 6.57)	4.00 × ${10}^{-16}$ (***)	5.55 (4.36, 7.11)	4.67 × ${10}^{-16}$ (***)	11.12 (7.75, 16.01)	4.00 × ${10}^{-16}$ (***)	10.04 (6.93, 14.60)	4.67 × ${10}^{-16}$ (***)
Sensory environment of the waiting room	6.42 (5.14, 8.07)	4.00 × ${10}^{-16}$ (***)	6.28 (4.97, 7.98)	4.67 × ${10}^{-16}$ (***)	9.70 (6.78, 13.94)	4.00 × ${10}^{-16}$ (***)	8.08 (5.61, 11.70)	4.67 × ${10}^{-16}$ (***)
Sensory environment of the office	6.98 (5.45, 9.02)	4.00 × ${10}^{-16}$ (***)	6.85 (5.29, 8.95)	4.67 × ${10}^{-16}$ (***)	10.02 (6.87, 14.64)	4.00 × ${10}^{-16}$ (***)	8.28 (5.62, 12.21)	4.67 × ${10}^{-16}$ (***)
Seeing a different healthcare professional to whom you expect	7.68 (5.97, 9.98)	4.00 × ${10}^{-16}$ (***)	8.03 (6.16, 10.57)	4.67 × ${10}^{-16}$ (***)	11.51 (7.89, 16.85)	4.00 × ${10}^{-16}$ (***)	9.59 (6.50, 14.21)	4.67 × ${10}^{-16}$ (***)
Talking to a healthcare professional	5.93 (4.73, 7.49)	4.00 × ${10}^{-16}$ (***)	6.32 (4.96, 8.11)	4.67 × ${10}^{-16}$ (***)	11.45 (7.98, 16.50)	4.00 × ${10}^{-16}$ (***)	9.56 (6.58, 13.96)	4.67 × ${10}^{-16}$ (***)
Picking up a prescription	5.21 (3.68, 7.53)	4.00 × ${10}^{-16}$ (***)	5.17 (3.59, 7.61)	4.67 × ${10}^{-16}$ (***)	8.74 (5.42, 14.13)	4.00 × ${10}^{-16}$ (***)	7.21 (4.40, 11.80)	7.28 × ${10}^{-15}$ (***)
Having to see many healthcare professionals before being able to talk to a specialist	5.19 (4.18, 6.48)	4.00 × ${10}^{-16}$ (***)	5.08 (4.05, 6.41)	4.67 × ${10}^{-16}$ (***)	8.86 (6.22, 12.68)	4.00 × ${10}^{-16}$ (***)	7.47 (5.20, 10.79)	4.67 × ${10}^{-16}$ (***)
After a diagnosis of any kind due to lack of follow-up or support	5.49 (4.47, 6.77)	4.00 × ${10}^{-16}$ (***)	5.47 (4.41, 6.81)	4.67 × ${10}^{-16}$ ( ***)	9.86 (6.94, 14.13)	4.00 × ${10}^{-16}$ (***)	8.64 (6.03, 12.47)	4.67 × ${10}^{-16}$ (***)
*Triggers for a meltdown*								
The idea of going to see a healthcare professional	4.56 (3.29, 6.44)	4.00 × ${10}^{-16}$ (***)	4.46 (3.17, 6.40)	4.67 × ${10}^{-16}$ (***)	8.09 (5.11, 12.80)	4.00 × ${10}^{-16}$ (***)	6.47 (4.04, 10.36)	1.39 × ${10}^{-14}$ (***)
Setting up an appointment to see a healthcare professional	4.44 (3.16, 6.37)	4.00 × ${10}^{-16}$ (***)	4.03 (2.83, 5.86)	1.10 × ${10}^{-13}$ (***)	8.67 (5.43, 13.83)	4.00 × ${10}^{-16}$ (***)	6.72 (4.16, 10.85)	1.24 × ${10}^{-14}$ (***)
Sensory environment of the waiting room	6.96 (4.84, 10.31)	4.00 × ${10}^{-16}$ (***)	6.60 (4.53, 9.87)	4.67 × ${10}^{-16}$ (***)	9.45 (5.71, 15.67)	4.00 × ${10}^{-16}$ (***)	7.67 (4.60, 12.84)	1.30 × ${10}^{-14}$(***)
Sensory environment of the office	6.43 (4.26, 10.09)	4.00 × ${10}^{-16}$ (***)	5.79 (3.78, 9.18)	1.32 × ${10}^{-14}$ (***)	8.01 (4.48, 14.29)	2.53 × ${10}^{-12}$ (***)	6.47 (3.58, 11.66)	2.69 × ${10}^{-17}$(***)
Seeing a different healthcare professional to whom you expect	6.04 (4.24, 8.83)	4.00 × ${10}^{-16}$ (***)	5.38 (3.74, 7.95)	4.67 × ${10}^{-16}$ (***)	9.84 (6.07, 16.02)	4.00 × ${10}^{-16}$ (***)	7.73 (4.72, 12.70)	1.05 × ${10}^{-15}$ (***)
Talking to a healthcare professional	6.30 (4.39, 9.30)	4.00 × ${10}^{-16}$ (***)	6.21 (4.28, 9.27)	4.67 × ${10}^{-16}$ (***)	6.37 (3.72, 10.83)	1.41 × ${10}^{-11}$ (***)	5.61 (3.25, 9.61)	6.06 × ${10}^{-10}$ (***)
Picking up a prescription	6.35 (3.91, 10.93)	2.27 × ${10}^{-12}$ (***)	5.10 (3.09, 8.87)	1.68 × ${10}^{-9}$ (***)	8.83 (4.56, 17.22)	1.45 × ${10}^{-10}$ (***)	6.61 (3.36, 13.09)	6.25 × ${10}^{-8}$ (***)
Having to see many healthcare professionals before being able to talk to a specialist	4.67 (3.59, 6.15)	4.00 × ${10}^{-16}$ (***)	4.61 (3.49, 6.14)	4.67 × ${10}^{-16}$ (***)	6.54 (4.36, 9.75)	4.00 × ${10}^{-16}$ (***)	5.33 (3.53, 8.03)	3.09 × ${10}^{-15}$ (***)
After a diagnosis of any kind due to lack of follow-up or support	5.05 (3.96, 6.50)	4.00 × ${10}^{-16}$ (***)	5.25 (4.05, 6.86)	4.67 × ${10}^{-16}$ (***)	8.22 (5.65, 11.96)	4.00 × ${10}^{-16}$ (***)	6.72 (4.57, 9.88)	4.67 × ${10}^{-16}$ (***)

*OR* odds ratio, *95% CI* 95% confidence interval, *Sig.* significance level

^a^Binomial Logistic Regression adjusting for age, sex, ethnicity, education level, and country of residence

*p*-value: < 0.05 = *; < 0.01 = **; < 0.001 = ***

**Table 3:** Self-reported healthcare experiences for transgender autistic adults compared to cisgender autistic adults

	Unadjusted		Adjusted model^a^	
	OR (95% CI)	*p*-value	OR (95% CI)	*p*-value
*General healthcare experience*				
Are you able to see healthcare professionals as often as you would like?	0.74 (0.53, 1.01)	0.12	0.69 (0.49, 0.96)	0.13
Do you have health insurance?	2.64 (1.61, 4.60)	2.08 × ${10}^{-3}$ (**)	2.31 (1.35, 4.20)	0.05 (*)
*Autism and healthcare*				
I have told my healthcare professional that I am autistic	0.92 (0.63, 1.37)	0.75	1.10 (0.73, 1.67)	0.71
My healthcare professional and I have discussed my autism	0.90 (0.64, 1.26)	0.66	0.90 (0.63, 1.28)	0.65
My healthcare professional knows what autism is	1.05 (0.74, 1.51)	0.82	1.15 (0.80, 1.67)	0.60
I think that my healthcare professional usually tries to make adjustments for me because I am autistic	1.10 (0.77, 1.56)	0.70	1.20 (0.83, 1.72)	0.48
I think that my healthcare professional usually considers my autism when making diagnoses and treatment plans	0.87 (0.59, 1.26)	0.59	0.87 (0.59, 1.28)	0.62
*Communication*				
I am usually able to explain what my symptoms are	0.68 (0.49, 0.95)	0.06	0.79 (0.56, 1.13)	0.36
I usually understand what my healthcare professional means when they discuss my health	0.62 (0.44, 0.89)	0.03 (*)	0.68 (0.48, 0.99)	0.17
I do not usually ask all the questions I would like to about my health	1.09 (0.74, 1.63)	0.76	0.92 (0.61, 1.40)	0.72
I can bring up a health concern even if my healthcare professional doesn’t ask about it	0.63 (0.46, 0.87)	0.02 (*)	0.78 (0.55, 1.10)	0.33
I know what is expected of me when I go to see my healthcare professional	0.48 (0.35, 0.67)	2.26 × ${10}^{-4}$ (***)	0.57 (0.40, 0.81)	0.04 (*)
*Anxiety*				
The idea of going to see a healthcare professional makes me feel anxious	1.63 (1.02, 2.74)	0.11	1.27 (0.77, 2.19)	0.52
The environment of the waiting room office makes me feel anxious	0.98 (0.66, 1.48)	0.93	0.74 (0.49, 1.15)	0.36
I feel anxious when I see a different healthcare professional to whom I expect	2.21 (1.31, 3.99)	0.02 (*)	1.76 (1.02, 3.25)	0.21
The process of setting up an appointment makes me anxious	2.20 (1.31, 3.97)	0.02 (*)	1.71 (0.99, 3.14)	0.24
The process of picking up a prescription makes me anxious	1.47 (1.05, 2.08)	0.06 (*)	1.25 (0.88, 1.79)	0.36
I frequently leave my healthcare professional’s office feeling as though I did not receive any help at all	0.94 (0.68, 1.32)	0.78	0.78 (0.55, 1.11)	0.35
*Access and advocacy*				
I know who to contact if I have a healthcare concern	0.64 (0.45, 0.92)	0.04 (*)	0.72 (0.50, 1.04)	0.24
If I need to go to see a healthcare professional, I am able to get there	0.41 (0.28, 0.58)	1.32 × ${10}^{-5}$ (***)	0.44 (0.30, 0.66)	3.37 × ${10}^{-3}$ (**)
I usually bring someone along to help support me in my appointments	1.44 (1.03, 2.00)	0.07	1.15 (0.80, 1.64)	0.59
If I need to go to the pharmacy, I am able to get there	0.47 (0.31, 0.72)	3.09 × ${10}^{-3}$ (**)	0.45 (0.29, 0.71)	0.01 (**)
I am able to follow a procedure for next steps if asked (for example, I will attend follow-up appointments, annual checkups if applicable, etc.…)	0.56 (0.39, 0.82)	0.01 (**)	0.65 (0.45, 0.97)	0.13
I am able to make appointments for myself	0.61 (0.42, 0.90)	0.04 (*)	0.73 (0.49, 1.11)	0.32
I will wait until it is an emergency before I go to see a healthcare professional	1.28 (0.91, 1.83)	0.25	1.25 (0.88, 1.81)	0.36
Chosen not to go in to see a healthcare professional regarding a health concern	2.26 (1.41, 3.83)	5.56 × ${10}^{-3}$ (**)	1.78 (1.09, 3.06)	0.13
*System*				
In most appointments, I have enough time to discuss my concerns with healthcare professionals	0.88 (0.63, 1.23)	0.57	0.91 (0.64, 1.28)	0.67
If I need to go to see a specialist for a healthcare concern, I am able to do so	0.58 (0.42, 0.81)	5.56 × ${10}^{-3}$ (**)	0.63 (0.45, 0.89)	0.09
I often choose not to go to the doctor with concerns if I need to see a specialist because I know that it will take me many appointments before I can see the specialist	1.48 (1.06, 2.08)	0.06	1.25 (0.88, 1.79)	0.36
I usually leave my appointments knowing what the next steps are (i.e. follow-up appointments, medications, etc)	0.77 (0.55, 1.08)	0.21	0.90 (0.63, 1.29)	0.65
I am provided with appropriate support after I receive a diagnosis of any kind (i.e. anything from infections to chronic conditions)	0.77 (0.54, 1.08)	0.23	0.80 (0.55, 1.14)	0.36
*Sensory experiences*				
Reported at least one sensory difference (hyper- or hyposensitivity)	3.44 (1.41, 11.39)	0.05 × ${10}^{-3}$ (*)	2.16 (0.86, 7.25)	0.33
I am able to describe how my symptoms feel in my body	0.77 (0.56, 1.06)	0.20	0.94 (0.67, 1.31)	0.74
I am able to describe how bad my pain feels	0.53 (0.38, 0.74)	1.47 × 10 (***)	0.66 (0.47, 0.93)	0.12
I am able to describe my sensory processing differences to healthcare professionals	0.80 (0.57, 1.12)	0.29	1.01 (0.71, 1.43)	0.96
The sensory environment of the waiting room is more overwhelming than other environments	1.04 (0.73, 1.49)	0.86	0.88 (0.61, 1.29)	0.62
The sensory environment of the office is more overwhelming than other environments	0.88 (0.64, 1.22)	0.57	0.81 (0.58, 1.14)	0.36
My senses frequently overwhelm me so that I have trouble focusing on conversations with healthcare professionals	1.51 (1.07, 2.16)	0.06	1.27 (0.88, 1.86)	0.36
*Triggers for a shutdowns*				
The idea of going to see a healthcare professional	1.60 (1.15, 2.22)	0.02 (*)	1.33 (0.94, 1.88)	0.28
Setting up an appointment to see a healthcare professional	2.14 (1.54, 2.98)	1.05 × ${10}^{-4}$ (***)	1.82 (1.29, 2.56)	0.02 (*)
Sensory environment of the waiting room	1.15 (1.09, 2.11)	0.04 (*)	1.34 (0.95, 1.89)	0.28
Sensory environment of the office	1.44 (1.03, 2.00)	0.08	1.26 (0.89, 1.79)	0.36
Seeing a different healthcare professional to whom you expect	1.50 (1.08, 2.09)	0.05 (*)	1.21 (0.85, 1.72)	0.45
Talking to a healthcare professional	1.93 (1.39, 2.69)	9.89 × ${10}^{-4}$ (***)	1.54 (1.08, 2.19)	0.12
Picking up a prescription	1.68 (1.12, 2.48)	0.20	1.41 (0.92, 2.12)	0.29
Having to see many healthcare professionals before being able to talk to a specialist	1.71 (1.23, 2.38)	0.01 (**)	1.49 (1.05, 2.11)	0.13
After a diagnosis of any kind due to lack of follow-up or support	1.80 (1.29, 2.53)	4.45 × ${10}^{-3}$ (**)	1.67 (1.18, 2.38)	0.05 (*)
*Triggers for a meltdown*				
The idea of going to see a healthcare professional	1.78 (1.19, 2.61)	0.02 (*)	1.46 (0.96, 2.18)	0.24
Setting up an appointment to see a healthcare professional	1.95 (1.31, 2.87)	5.34 × ${10}^{-3}$ (**)	1.63 (1.07, 2.45)	0.12
Sensory environment of the waiting room	1.36 (0.90, 2.01)	0.23	1.17 (0.76, 1.76)	0.61
Sensory environment of the office	1.25 (0.77, 1.95)	0.49	1.13 (0.68, 1.81)	0.70
Seeing a different healthcare professional to whom you expect	1.63 (1.09, 2.39)	0.05 (*)	1.46 (0.96, 2.19)	0.24
Talking to a healthcare professional	1.01 (0.64, 1.56)	0.96	0.90 (0.56, 1.40)	0.70
Picking up a prescription	1.39 (0.81, 2.28)	0.30	1.27 (0.73, 2.15)	0.53
Having to see many healthcare professionals before being able to talk to a specialist	1.40 (0.97, 1.99)	0.13	1.15 (0.79, 1.67)	0.60
After a diagnosis of any kind due to lack of follow-up or support	1.63 (1.16, 2.28)	0.02 (*)	1.27 (0.89, 1.81)	0.36

*OR* odds ratio, *95% CI* 95% confidence interval, *Sig.* significance level

^a^Binomial Logistic Regression adjusting for age, sex, ethnicity, education level, and country of residence

*p*-value: < 0.05 = *; < 0.01 = **; < 0.001 = ***

### Overall physical and mental health outcomes

**Fig. 1:**
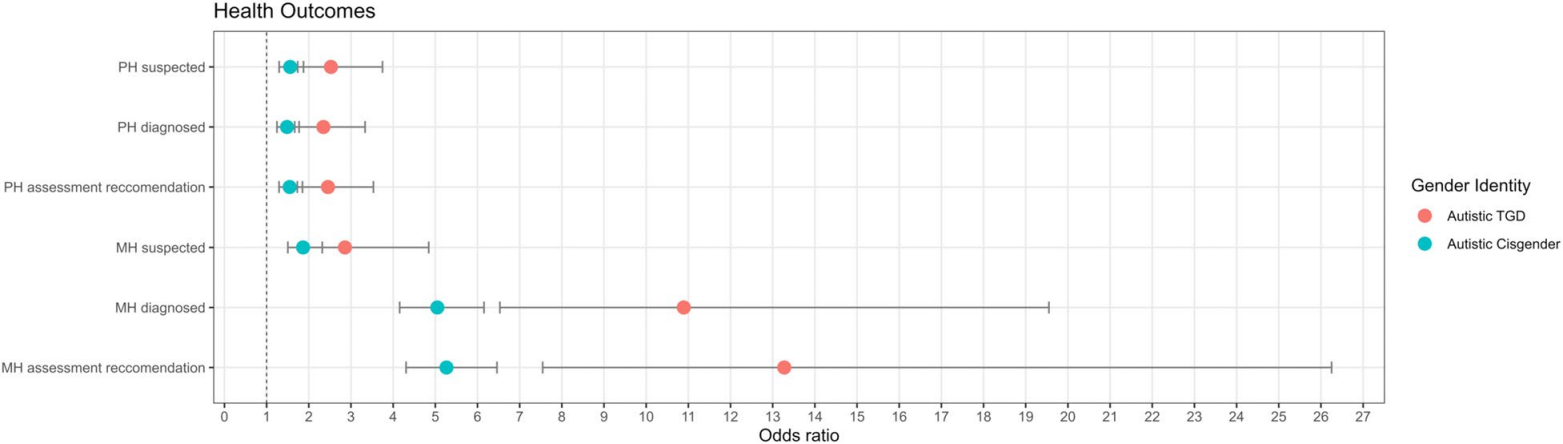
Odds ratios of diagnosed, suspected, and assessment recommendations for mental health (MH) and physical health (PH) conditions for cisgender and TGD autistic individuals compared to cisgender non-autistic individuals. *MH* mental health, *PH* physical health, *TGD* transgender/gender diverse

Additionally, mental and physical health conditions (including conditions that are formally diagnosed, suspected, or recommended for assessment) were significantly more frequent among cisgender autistic and TGD autistic individuals compared to cisgender non-autistic individuals. For every 10 cisgender non-autistic individuals who reported a diagnosed physical health condition, 15 cisgender autistic individuals (AOR = 1.48, 95% CI 1.25, 1.77) and 23 TGD autistic individuals reported the same (AOR = 2.35, 95% CI 1.67, 3.34). For every 10 cisgender non-autistic individuals who reported a diagnosed mental health condition, 50 cisgender autistic individuals (AOR = 5.05, 95% CI 4.16, 6.16) and 109 TGD autistic individuals reported the same (AOR = 10.89, 95% CI 6.53, 19.55). Disparities were also found for mental and physical health conditions that were suspected or that individuals were recommended assessments for between cisgender non-autistic individuals and cisgender autistic and TGD autistic individuals, as shown in Table 4 and Fig. 1.

**Table 4:** Self-reported health outcomes for transgender/gender diverse and cisgender autistic adults compared to cisgender non-autistic adults

	Cisgender autistic adults				Transgender/gender diverse autistic adults			
	Unadjusted model		Adjusted model^a^		Unadjusted model		Adjusted model^a^	
	OR (95% CI)	*p*-value (sig.)	OR (95% CI)	*p*-value (sig.)	OR (95% CI)	*p*-value (sig.)	OR (95% CI)	*p*-value (sig.)
*Physical health conditions*								
Rates of diagnosed conditions	1.690 (1.44, 2.87)	4.48 × ${10}^{-10}$ (***)	1.48 (1.25, 1.77)	1.38 × ${10}^{-5}$ (***)	2.05 (1.47, 2.87)	2.94 × ${10}^{-5}$ (***)	2.35 (1.67, 3.34)	1.90 × ${10}^{-6}$ (***)
Rates of suspected conditions	1.71 (1.44, 2.04)	1.44 × ${10}^{-9}$ (***)	1.56 (1.30, 1.88)	2.94 × ${10}^{-6}$ (***)	2.30 (1.60, 3.38)	1.50 × ${10}^{-5}$ (***)	2.53 (1.74, 3.75)	2.86 × ${10}^{-6}$ (***)
Rates of condition assessment recommendations	1.71 (1.45, 2.03)	2.78 × ${10}^{-10}$ (***)	1.55 (1.30, 1.85)	2.07 × ${10}^{-6}$ (***)	2.30 (1.53, 2.03)	1.63 × ${10}^{-5}$ (***)	2.46 (1.73, 3.53)	1.09 × ${10}^{-6}$ (***)
*Specific physical health conditions*								
Arthritis/ongoing back or joint problems	1.79 (1.47, 2.18)	7.83 × ${10}^{-9}$ (***)	1.63 (1.32, 2.01)	6.49 × ${10}^{-6}$ (***)	1.98 (1.38, 2.82)	1.94 × ${10}^{-4}$ (***)	2.27 (1.55, 3.31)	2.82 × ${10}^{-5}$ (***)
Blindness/partial sight	0.80 (0.50, 1.28)	0.38	1.03 (0.62, 1.69)	0.91	0.50 (0.12, 1.39)	0.26	0.57 (0.14, 1.62)	0.38
Breathing conditions	1.56 (1.28, 1.90)	1.47 × ${10}^{-5}$ (***)	1.50 (1.22, 1.85)	1.19 × ${10}^{-4}$ (***)	1.98 (1.38, 2.82)	1.76 × ${10}^{-4}$ (***)	1.95 (1.36, 2.79)	3.01 × ${10}^{-4}$ (***)
Cancer	0.75 (0.50, 1.11)	0.17	0.80 (0.52, 1.21)	0.31	0.57 (0.20, 1.30)	0.24	0.83 (0.28, 2.05)	0.73
Deafness or hearing loss	1.881 (1.358, 2.623)	1.89 × ${10}^{-4}$ (***)	1.705 (1.214, 2.407)	0.00 (**)	1.067 (0.487, 2.078)	0.88	1.360 (0.61, 2.70)	0.44
Diabetes	1.84 (1.26, 2.72)	0.00 (**)	1.74 (1.17, 2.62)	0.01 (**)	1.34 (0.58, 2.74)	0.48	1.79 (0.75, 3.8)	0.16
Heart conditions	1.99 (1.45, 2.74)	2.59 × ${10}^{-5}$ (***)	1.95 (1.41, 2.72)	8.02 × ${10}^{-5}$ (***)	1.24 (0.61, 2.30)	0.55	1.68 (0.81, 3.18)	0.15
High blood pressure	1.22 (0.96, 1.55)	0.11	1.13 (0.87, 1.48)	0.37	1.01 (0.61, 1.61)	0.98	1.76 (1.01, 2.94)	0.04 (*)
Intellectual disability	3.75 (2.20, 6.72)	3.61 × ${10}^{-6}$ (***)	2.71 (1.54, 4.97)	9.02 × ${10}^{-4}$ (***)	4.10 (1.72, 9.15)	8.79 × ${10}^{-4}$ (***)	3.48 (1.42, 8.03)	0.00 (**)
Kidney or liver disease	1.39 (0.89, 2.18)	0.16	1.62 (1.02, 2.60)	0.04 (*)	1.01 (0.34, 2.37)	1.00	1.45 (0.49, 2.60)	0.46
Neurological condition	2.04 (1.44, 2.92)	9.42 × ${10}^{-5}$ (***)	2.11 (1.47, 3.06)	7.98 × ${10}^{-5}$ (***)	5.18 (3.19, 8.31)	2.17 × ${10}^{-11}$ (***)	5.27 (3.20, 8.55)	4.58 × ${10}^{-11}$ (***)
*Mental health conditions*								
Rates of diagnosed conditions	4.86 (4.04, 5.86)	4.00 × ${10}^{-16}$ (***)	5.05 (4.16, 6.16)	4.67 × ${10}^{-16}$ (***)	12.02 (7.24, 21.50)	4.00 × ${10}^{-16}$ (***)	10.89 (6.53, 19.55)	4.67 × ${10}^{-16}$ (***)
Rates of suspected conditions	1.99 (1.62, 2.45)	1.19 × ${10}^{-10}$ (***)	1.87 (1.50, 2.32)	2.43 × ${10}^{-8}$ (***)	2.88 (1.62, 4.86)	3.00 × ${10}^{-5}$ (***)	2.86 (1.78, 4.85)	4.35 × ${10}^{-5}$ (***)
Rates of condition assessment recommendations	5.04 (4.16, 6.13)	4.00 × ${10}^{-16}$ (***)	5.27 (4.31, 6.46)	4.67 × ${10}^{-16}$ (***)	14.70 (8.29, 29.0)	4.00 × ${10}^{-16}$ (***)	13.27 (7.46, 26.25)	9.74 × ${10}^{-16}$ (***)
*Specific mental health conditions*								
Anorexia nervosa	3.40 (2.30, 5.15)	3.17 × ${10}^{-9}$ (***)	3.60 (2.39, 5.55)	3.03 × ${10}^{-9}$ (***)	5.08 (2.84, 8.92)	2.83 × ${10}^{-8}$ (***)	3.99 (2.20, 7.09)	3.95 × ${10}^{-6}$ (***)
Anxiety	3.86 (3.26, 4.58)	4.00 × ${10}^{-16}$ (***)	3.84 (3.21, 4.60)	4.67 × ${10}^{-16}$ (***)	6.00 (4.25, 8.61)	4.00 × ${10}^{-16}$ (***)	5.24 (3.69, 7.56)	4.67 × ${10}^{-16}$ (***)
ADHD	3.27 (2.43, 4.44)	2.04 × ${10}^{-14}$ (***)	4.82 (3.50, 6.71)	4.67 × ${10}^{-16}$ (***)	5.47 (3.52, 8.41)	3.40 × ${10}^{-14}$ (***)	6.73 (4.24, 10.60)	7.13 × ${10}^{-16}$ (***)
Binge eating	1.80 (1.17, 2.80)	0.01 (**)	1.82 (1.16, 2.89)	0.01 (**)	1.29 (0.48, 2.89)	0.60	1.10 (0.41, 2.51)	0.83
Bipolar disorder	2.200 (1.453, 3.384)	2.76 × ${10}^{-4}$ (***)	2.377 (1.541, 3.724)	1.34 × ${10}^{-4}$ (***)	3.396 (1.766, 6.243)	1.54 × ${10}^{-4}$ (***)	3.48 (1.78, 6.51)	1.77 × ${10}^{-4}$ (***)
Bulimia	3.23 (1.87, 5.82)	5.57 × ${10}^{-5}$ (***)	3.51 (1.99, 6.50)	3.64 × ${10}^{-5}$ (***)	2.69 (0.96, 6.56)	0.04 (*)	2.09 (0.74, 5.18)	0.14
Depression	3.70 (3.12, 4.38)	4.00 × ${10}^{-16}$ (***)	3.61 (3.03, 4.32)	4.67 × ${10}^{-16}$ (***)	5.20 (3.69, 7.44)	4.00 × ${10}^{-16}$ (***)	4.89 (3.45, 7.03)	4.67 × ${10}^{-16}$ (***)
Insomnia	2.58 (2.06, 3.25)	5.02 × ${10}^{-16}$ (***)	3.01 (2.37, 3.83)	4.67 × ${10}^{-16}$ (***)	3.56 (2.44, 5.14)	3.34 × ${10}^{-11}$ (***)	3.63 (2.47, 5.30)	5.86 × ${10}^{-11}$ (***)
OCD	6.08 (4.21, 9.03)	4.00 × ${10}^{-16}$ (***)	6.62 (4.50, 10.00)	4.67 × ${10}^{-16}$ (***)	6.52 (3.78, 11.13)	1.39 × ${10}^{-11}$ (***)	5.65 (3.24, 9.78)	1.09 × ${10}^{-9}$ (***)
Panic disorder	2.59 (1.92, 3.51)	8.19 × ${10}^{-10}$ (***)	3.02 (2.21, 4.16)	1.27 × ${10}^{-11}$ (***)	4.16 (2.63, 6.48)	7.46 × ${10}^{-10}$ (***)	4.13 (2.57, 6.56)	4.12 × ${10}^{-9}$ (***)
Personality disorder	4.82 (3.27, 7.29)	2.05 × ${10}^{-14}$ (***)	4.77 (3.20, 7.32)	2.31 × ${10}^{-13}$ (***)	5.13 (2.82, 9.12)	5.12 × ${10}^{-8}$ (***)	4.01 (2.18, 7.23)	6.31 × ${10}^{-6}$ (***)
PTSD	3.23 (2.47, 4.25)	4.00 × ${10}^{-16}$ (***)	3.68 (2.78, 4.91)	4.67 × ${10}^{-16}$ (***)	5.72 (3.83, 8.50)	4.00 × ${10}^{-16}$ (***)	5.67 (3.74, 8.53)	4.67 × ${10}^{-16}$ (***)
Postnatal depression	1.74 (1.19, 2.58)	0.00 (**)	1.70 (1.14, 2.55)	0.01 (*)	0.80 (0.28, 1.87)	0.66	0.72 (0.24, 1.71)	0.51
Schizophrenia	6.28 (2.61, 18.62)	2.01 × ${10}^{-4}$ (***)	7.24 (2.94, 21.82)	9.17 × ${10}^{-5}$ (***)	4.53 (0.92, 18.61)	0.04 (*)	4.89 (0.97, 20.70)	0.03 (*)
Seasonal affective disorder	3.07 (2.04, 4.73)	1.92× ${10}^{-7}$ (***)	3.17 (2.07, 4.94)	2.4 × ${10}^{-7}$ (***)	4.27 (2.27, 7.78)	4.00 × ${10}^{-6}$ (***)	4.35 (2.29, 8.02)	4.63 × ${10}^{-6}$ (***)
Self-harm	4.10 (3.17, 5.35)	4.00 × ${10}^{-16}$ (***)	4.58 (3.70, 6.09)	4.67 × ${10}^{-16}$ (***)	7.59 (5.18, 11.10)	4.00 × ${10}^{-16}$ (***)	5.84 (3.92, 8.69)	4.67 × ${10}^{-16}$ (***)

*OR* odds ratio, *95% CI* 95% confidence interval, *Sig.* significance level

^a^Binomial Logistic Regression adjusting for age, sex, ethnicity, education level, and country of residence

*p*-value: < 0.05 = *; < 0.01 = **; < 0.001 = ***

### Mental and physical health condition outcomes for individual conditions

Regarding specific conditions, compared to cisgender non-autistic adults, both TGD autistic and cisgender autistic people had significantly higher rates of arthritis, breathing conditions, intellectual disability, neurological conditions, anorexia, anxiety, ADHD, bipolar, depression, insomnia, OCD, panic disorder, personality disorders, post-traumatic stress disorder (PTSD), schizophrenia, seasonal affective disorder (SAD), and self-harm. TGD autistic individuals were also uniquely more likely to have high blood pressure, whereas cisgender autistic individuals had uniquely high rates of deafness/hearing loss, diabetes, heart conditions, kidney or liver disease, binge-eating, bulimia, and post-natal depression. When cisgender autistic and TGD autistic adults were compared directly, TGD autistic adults were significantly more likely to have a neurological condition; no other significant differences were found. Full results can be found in Tables 4 and 5.

**Table 5:** Self-reported health outcomes for cisgender autistic adults compared to transgender/gender diverse autistic adults

	Unadjusted model		Adjusted model^a^	
	OR (95% CI)	*p*-value (sig.)	OR (95% CI)	*p*-value (sig.)
*Physical health conditions*				
Rates of diagnosed conditions	0.83 (0.59, 1.15)	0.38	0.66 (0.46, 0.94)	0.12
Rates of suspected conditions	0.74 (0.50, 1.08)	0.22	0.65 (0.44, 0.96)	0.13
Rates of condition assessment recommendations	0.74 (0.56, 1.13)	0.30	0.65 (0.45, 0.94)	0.12
*Specific physical health conditions*				
Arthritis/ongoing back or joint problems	0.90 (0.64, 1.29)	0.67	0.73 (0.50, 1.08)	0.29
Blindness/partial sight	1.61 (0.57, 6.75)	0.57	1.86 (0.63, 8.00)	0.48
Breathing conditions	0.78 (0.55, 1.12)	0.27	0.79 (0.55, 1.14)	0.36
Cancer	1.32 (0.56, 3.85)	0.67	0.94 (0.36, 2.98)	0.93
Deafness or hearing loss	1.76 (0.92, 3.82)	0.20	1.32 (0.67, 2.91)	0.60
Diabetes	1.38 (0.69, 3.15)	0.55	0.97 (0.46, 2.27)	0.94
Heart conditions	1.61 (0.88, 3.23)	0.23	1.16 (0.62, 2.38)	0.71
High blood pressure	1.21 (0.77, 2.02)	0.57	0.69 (0.41, 1.19)	0.35
Intellectual disability	0.92 (0.46, 2.02)	0.84	0.77 (0.37, 1.77)	0.63
Kidney or liver disease	1.38 (0.59, 4.04)	0.61	1.27 (0.53, 3.81)	0.70
Neurological condition	0.39 (0.25, 0.62)	4.71 × ${10}^{-4}$ (***)	0.39 (0.25, 0.63)	3.37 × ${10}^{-3}$ (**)
*Mental health conditions*				
Rates of diagnosed conditions	0.40 (0.22, 0.68)	0.00 (**)	0.48 (0.26, 0.82)	0.09
Rates of suspected conditions	0.69 (0.40, 1.12)	0.23	0.64 (0.37, 1.04)	0.27
Rates of condition assessment recommendations	0.34 (0.17, 0.62)	0.00 (***)	0.40 (0.20, 0.73)	0.09
*Specific mental health conditions*				
Anorexia nervosa	0.67 (0.41, 1.13)	0.21	0.91 (0.55, 1.58)	0.76
Anxiety	0.64 (0.45, 0.91)	0.05 (*)	0.72 (0.50, 1.04)	0.25
ADHD	0.60 (0.41, 0.89)	0.03 (*)	0.71 (0.47, 1.09)	0.30
Binge eating	1.40 (0.64, 3.68)	0.57	1.57 (0.70, 4.19)	0.48
Bipolar disorder	0.65 (0.37, 1.21	0.23	0.74 (0.41, 1.40)	0.48
Bulimia	1.20 (0.54, 3.18)	0.75	1.66 (0.73, 4.47)	0.43
Depression	0.71 (0.50, 1.01)	0.12	0.75 (0.52, 1.07)	0.30
Insomnia	0.73 (0.51, 1.04)	0.16	0.83 (0.58, 1.21)	0.48
OCD	0.93 (0.60, 1.49)	0.81	1.15 (0.73, 1.87)	0.65
Panic disorder	0.62 (0.41, 0.96)	0.07	0.72 (0.47, 1.14)	0.33
Personality disorder	0.94 (0.58, 1.60)	0.84	1.20 (0.73, 2.07)	0.62
PTSD	0.56 (0.39, 0.82)	0.00 (**)	0.63 (0.44, 0.93)	0.12
Postnatal depression	2.17 (0.95, 6.27)	0.19	2.39 (1.02, 7.01)	0.24
Schizophrenia	1.39 (0.48, 5.86)	0.68	1.53 (0.51, 6.62)	0.62
Seasonal affective disorder	0.72 (0.42, 1.28)	0.34	0.70 (0.41, 1.27)	0.36
Self-harm	0.54 (0.39, 0.76)	0.00 (***)	0.75 (0.53, 1.09)	0.31

*OR* odds ratio, *95% CI* 95% confidence interval, *Sig.* significance level

^a^Binomial Logistic Regression adjusting for age, sex, ethnicity, education level, and country of residence

*p*-value: < 0.05 = *; < 0.01 = **; < 0.001 = ***

## Discussion

Stark differences in self-reported healthcare experiences were found among cisgender autistic and TGD autistic adults compared to cisgender non-autistic adults across 50/51 items. Poorer healthcare experiences span across the domains of general healthcare experiences, communication, anxiety, access and advocacy, system-level problems, sensory experiences, shutdowns, and meltdowns. Both cisgender autistic and TGD autistic adults had alarmingly high rates of mental health conditions (including conditions that are formally diagnosed, suspected, or recommended for assessment) and self-harm compared to cisgender non-autistic adults. Cisgender autistic people were 4.6 times and TGD autistic people were 5.8 times more likely to report self-harm than cisgender non-autistic individuals, respectively. Our results also suggest that there are at least five key areas in which the needs of TGD autistic adults are uniquely less likely to be met, even compared to cisgender autistic adults.

Challenges in accessing healthcare are likely to be multifactorial, and this may be particularly true for individuals with marginalised, intersectional identities. This was echoed by members of our focus group who felt the identified disparities could be a result of both TGD-related difficulties and autistic traits. Previous qualitative literature identifies that autistic TGD individuals report unique prejudice as a result of their intersecting identities, including from healthcare professionals, due to a lack of knowledge and inaccurate assumptions about autistic people’s ability to understand their own gender identity [36, 54, 58]. Our finding that TGD autistic individuals report additional barriers to getting to healthcare appointments and the pharmacy compared to cisgender autistic individuals may at least partly be explained by this difficulty accessing supportive and knowledgeable practitioners [35, 59], as well as the scarcity of clinics offering transgender-specific healthcare [40, 58]. These system and practitioner-level barriers to access may be exacerbated by the difficulties that TGD autistic individuals describe when trying to self-advocate around their gender needs, including difficulties correcting pronouns and explaining their gender in a way that others understand [60].

Acknowledging that communication is a dyadic process, the ‘double empathy problem’ describes the bidirectional breakdown in reciprocity between individuals who have contrasting dispositional ways of experiencing the world [61]. This has broadly been tested regarding interactions between autistic and non-autistic individuals; however recently, the ‘triple empathy’ problem has been used to describe how interaction difficulties experienced by autistic individuals may be compounded within healthcare contexts where patients with lay knowledge and doctors with expert knowledge struggle to understand each other’s perspectives [62]. It is possible that an intersecting TGD identity uniquely contributes to these communication challenges, since TGD patients may also experience the world differently from their presumably mostly cisgender healthcare providers [63, 64] as a result of their contrasting social realities in a cisnormative society. As the present study only collected data from patients and not healthcare providers, it cannot assess the applicability of these potential explanations; thus, future research should consider both perspectives.

The poor healthcare experiences relating to sensory sensitivities reported by both cisgender and TGD autistic individuals aligns with prior literature suggesting autistic people often find sound levels, lighting, and the proximity to other people in healthcare settings aversive or stress-provoking [65, 66]. Difficulties navigating new health care environments were also discussed by members of our focus group. This may also contribute to cisgender and TGD autistic individual’s abilities to communicate with professionals in healthcare settings [65] as well as increase the likelihood of shutdowns or meltdowns. Rates of diagnosed, suspected, and assessment recommendations for physical and mental health conditions were significantly greater in both cisgender autistic and TGD autistic individuals compared to cisgender non-autistic individuals. These results extend previous findings of health inequalities faced by these groups and are novel in their finding of the largest disparities between cisgender non-autistic and TGD autistic adults regarding mental health conditions (rather than physical health conditions).

While the increased risk of mental health conditions is likely multifactorial in nature (both between and within individuals), health disparities may be associated with minority stress: high levels of stress experienced by stigmatised minority members. Both autistic and TGD individuals are more likely to report prejudice, stigmatisation, discrimination, and concealment of identity [67–70]; they are also each more likely to report stressful/traumatic life events, such as bullying, harassment, abuse, victimisation, and exclusion [71–77]. While each of these factors have been individually associated with risk of health conditions [67–70, 72–77], the accumulation of these events across time can have a profound, negative impact on physical and mental health [78, 79]. Complex interactions between conditions and past experiences further increase the risk of additional health conditions, and indirect effects may also occur via coping behaviours that increase after minority stress exposure (e.g., substance use) [31, 80]. Minority stress and poor healthcare experiences, alongside intersectionality, may also explain why health outcome disparities were found to be the largest between cisgender non-autistic and TGD autistic individuals as having multiple stigmatised, minority identities, namely autistic and TGD identities, may result in even further isolation, distress, unmet healthcare needs, and mental health burden [81–85]. Higher rates of health conditions may also result from unmet healthcare needs potentially precipitated by poor quality self-reported healthcare. Several shared barriers to healthcare have been previously identified for both autistic and TGD adults such as lack of provider awareness/education/flexibility, difficulties communicating with practitioners, stigma, and discrimination; in addition to unique challenges for autistic individuals, such as difficulties with navigating the healthcare system, sensory sensitivities, bodily awareness, information processing, and diagnostic overshadowing; and unique challenges for TGD individuals, such as a shortage of specialist centres and issues with recording gender and names on data systems [19, 35–45]. Such experiences may lead to distrust and dissatisfaction with healthcare, reluctance to seek healthcare, and an increased likelihood of conditions going poorly managed or untreated, exacerbating poor health outcomes [37, 46, 86, 87].

## Limitations

Whilst this study is the first large-scale study to quantify health and healthcare disparities based on both autistic identity and gender identity, it also has limitations. Data were not originally collected with the aim of exploring associations between TGD identities and healthcare, and there was a relatively small TGD sample. As a result, the study did not include a TGD non-autistic comparison group, preventing us from drawing conclusions about whether the poor healthcare experiences and health conditions identified are unique to autistic TGD individuals or would be similar for non-autistic TGD groups. The small size of our TGD autistic sample also likely resulted in underpowered analyses (particularly regarding direct comparisons between the TGD and cisgender autistic groups). Further, our questionnaire item on gender identity did not capture the heterogenous individual identities among TGD individuals in detail. Since it only allowed participants to select ‘male’, ‘female’, ‘non-binary’ or ‘other’, individuals who identify as transgender may have selected ‘other’. Additionally, it was not possible to assess rates of health conditions among individuals with differing TGD identities, even though previous studies have found that rates may vary based on this [29, 31, 79, 88].

Another limitation is some items in our healthcare experiences survey may have been more likely to be endorsed by autistic individuals since they relate to common autistic traits/experiences, for example items on shutdowns, meltdowns and sensory sensitivities. In addition, our study failed to explore some themes identified by the focus group with TGD and cisgender autistic adults, which was conducted after data analysis to inform our interpretations of the results. A key theme was choice around modality of appointments. Some described difficulties waiting for phone call appointments at unspecified times, waiting in phone queues, and describing symptoms over the phone. In contrast, others described remote appointments as a vital option which reduces stress and sensory difficulties. Unfortunately, this was unexplored in the current study. Future research should consider how the modality of appointments may impact healthcare experiences and continue to collaborate with autistic and TGD individuals from the beginning of the study design process to ensure that the most relevant healthcare issues are addressed. It should also aim to use larger, representative samples of autistic TGD adults to facilitate a more in-depth interpretation of results and to reduce the likelihood of important disparities being missed due to underpowered analyses.

Generalisability of the study’s results are also limited by sampling biases, due to the nature of recruitment and the resulting demographics of the participants. This study utilised existing data from a larger self-report survey collected via convenience sampling that investigated chronic health conditions and healthcare experiences, meaning the sample may be biased towards those with relatively more health problems, more severe health problems, and those with negative healthcare experiences. Compared to the general population, the overall sample was biased towards white individuals, UK residents, highly educated individuals, those assigned female at birth, and those who currently identify as female. As such, our findings may be less likely to represent the experiences of people with different demographics, and of those with relatively good health. Due to low participation by intersex individuals (< 5 people), their responses had to be excluded from the study to conduct statistical analyses; as such, our findings may not accurately represent the experiences of intersex individuals. Participants who suspected autism, were self-diagnosed, or were waiting assessment were also excluded, meaning findings may not apply to these individuals who are not expected to be receiving reasonable adjustments relating to autism in their healthcare. The study’s design, as a lengthy online survey, also likely precluded the inclusion of some autistic individuals with intellectual disabilities.

There are further limitations regarding the type of data collected. Results refer to patients’ perceptions of healthcare [19], rather than objective measures of experiences (e.g., referral and prescribing patterns). Further, whilst the questionnaire addressed some provider-level factors, no data were collected from healthcare providers themselves. Future research should aim to include objective measures, alongside subjective measures of healthcare experiences collected from both patients and providers.

Nonetheless, the study’s strengths lie in its diverse international sample, and that it is the largest study to date comparing the health and healthcare experiences of cisgender and TGD autistic adults and cisgender non-autistic adults.

## Conclusions

Regardless of gender identity, autistic individuals have poorer self-reported healthcare experiences and increased health risks, particularly regarding mental health. Individuals who are transgender/gender diverse and autistic have additional challenges in accessing healthcare compared to both cisgender non-autistic and cisgender autistic individuals. While the present study provides important information about the health and healthcare experiences of transgender/gender diverse autistic people, more research is urgently needed in this area; in particular, future research should utilize generalizable samples to compare the experiences of transgender/gender diverse autistic people to cisgender autistic and non-autistic people, as well as transgender/gender diverse non-autistic people. Current healthcare systems must prioritise equity in healthcare for transgender/gender diverse autistic individuals. Clinical practice must recognise the unique challenges faced by transgender/gender diverse autistic adults and must prioritise needs-based and individualised care.

## Supplementary Information

Supplementary Material 1.

## Data Availability

We can provide group level data but not the underlying material itself, as our participants did not consent to having their data shared publicly. Underlying, anonymized data will be stored until 2026 and will only be made available to potential collaborators with ethical approval, after they submit a research proposal to the Autism Research Centre, University of Cambridge, UK, as is required by our original ethics application and participant consent form.
