# Supplementary material for: Autistic and transgender/gender diverse people’s experiences of health and healthcare
Source: Mol Autism. 2025 Jan 21;16:4. doi: 10.1186/s13229-024-00634-0 (PMC11748291; doi:10.1186/s13229-024-00634-0)
Supplement: Supplementary file 1 — Supplementary Material 1. [file 13229_2024_634_MOESM1_ESM.docx]

**Contents**

Supplementary tables………………………………………………………………………………..2

Table S1 Autistic participant demographic data only………………………………………2

Table S2 Missing participants per survey item for cisgender autistic and TGD autistic

compared to cisgender non-autistic individuals…………………………………………… 4

Table S3 Missing participants per survey item for cisgender autistic compared to TGD autistic

individuals……………………………………………………………….………………….9

**Table S1:** Demographics for autistic participants only

| Characteristics | Cisgender autistic  (n=1094) | Transgender  autistic (n=174) | p-values  (Sig.) |
| --- | --- | --- | --- |
| Age (years), mean (SD) | 42.12 (14.40) | 35.592 (13.91) | 2.96 x ${10}^{-8}$ (***) |
| Age (years), categories, N (%) |  |  |  |
| 16-29 | 262 (23.95) | 73 (41.95) |  |
| 30-39 | 226 (20.66) | 41 (23.56) |  |
| 40-49 | 240 (21.94) | 28 (16.09) |  |
| 50-59 | 229 (20.93) | 22 (12.64) |  |
| 60-69 | 106 (9.69) | 5 (2.87) |  |
| 70+ | 31 (2.83) | 5 (2.87) |  |
| AQ-10 Score, mean (SD) | 7.928 (1.90) | 8.512 (1.66) | 1.36 x ${10}^{-4}$ **(***)** |
| Biological Sex, N (%) |  |  | 4.96 x ${10}^{-8}$ (***) |
| Female | 665 (60.79) | 143 (82.18) |  |
| Male | 429 (39.21) | 31 (17.83) |  |
| Current gender identity, N (%) |  |  | 3.30 x ${10}^{-215}$ (***) |
| Female | 665 (60.79) | 8 (4.60) |  |
| Male | 429 (39.21) | 26 (14.94) |  |
| Other | 0 | 20 (11.49) |  |
| Non-Binary | 0 | 120 (68.97) |  |
| Ethnicity, N (%) |  |  | 0.06 |
| White | 950 (86.84) | 142 (81.61) |  |
| Non-white | 144 (13.16) | 32 (18.39) |  |
| African | 5 (0.46) | 0 (0) |  |
| Arab | 1 (0.09) | 0 (0) |  |
| Asian | 9 (0.82) | 0 (0) |  |
| Bangladeshi, Indian, Pakistani | 8 (0.73) | 2 (1.15) |  |
| Caribbean | 6 (0.55) | 1 (0.58) |  |
| Hispanic | 11 (1.01) | 0 (0) |  |
| Jewish | 24 (2.19) | 1 (0.58) |  |
| Turkish | 1 (0.09) | 0 (0) |  |
| Mixed race | 53 (4.85) | 23 (13.22) |  |
| Other | 26 (2.38) | 5 (2.87) |  |
| Country of residence, N (%) |  |  | 2.62 x ${10}^{-5}$ (***) |
| UK | 740 (67.64) | 90 (51.72) |  |
| USA | 107 (9.78) | 34 (19.54) |  |
| Other | 247 (22.58) | 50 (28.74) |  |
| Australia | 14 (1.28) | 6 (3.45) |  |
| Canada | 32 (2.93) | 8 (4.60) |  |
| Germany | 29 (2.65) | 10 (5.75) |  |
| Netherlands | 21 (1.92) | 4 (2.30) |  |
| Other | 151 (13.80) | 22 (12.64) |  |
| Education, N (%) |  |  | 0.03 (*) |
| No formal education | 47 (4.30) | 10 (5.75) |  |
| Secondary School/High School | 173 (15.81) | 43 (24.71) |  |
| Further vocational qualifications | 176 (16.09) | 24 (13.79) |  |
| University undergraduate | 352 (32.18) | 43 (24.71) |  |
| University postgraduate | 346 (31.63) | 54 (31.03) |  |

SD = standard deviation

p-values from Pearson’s Chi-Square test (for categorical variables) or Mann–Whitney U test (means for continuous variables)

Sig. = significance level

p-value: < .05 = * ; < .01 = ** ; < .001 = ***

**Table S2:** Missing participants per survey item for cisgender autistic compared to TGD autistic adults

| **Survey item** | **Number of participants with missing data** |
| --- | --- |
| **Healthcare experiences items** | |
| ***General healthcare experience*** | |
| Are you able to see healthcare professionals as often as you would like? | 27 |
| Do you have health insurance? | 5 |
| ***Autism and Healthcare*** | |
| I have told my healthcare professional that I am autistic | 73 |
| My healthcare professional and I have discussed my autism | 74 |
| My healthcare professional knows what autism is | 82 |
| I think that my healthcare professional usually tries to make adjustments for me because I am autistic | 84 |
| I think that my healthcare professional usually considers my autism when making diagnoses and treatment plans | 86 |
| ***Communication*** | |
| I am usually able to explain what my symptoms are | 18 |
| I usually understand what my healthcare professional means when they discuss my health | 17 |
| I do not usually ask all the questions I would like to about my health | 20 |
| I can bring up a health concern even if my healthcare professional doesn’t ask about it | 20 |
| I know what is expected of me when I go to see my healthcare professional | 17 |
| ***Anxiety*** | |
| The idea of going to see a healthcare professional makes me feel anxious | 24 |
| The environment of the waiting room office makes me feel anxious | 26 |
| I feel anxious when I see a different healthcare professional to whom I expect | 25 |
| The process of setting up an appointment makes me anxious | 26 |
| The process of picking up a prescription makes me anxious | 29 |
| I frequently leave my healthcare professional’s office feeling as though I did not receive any help at all | 25 |
| ***Access and advocacy*** | |
| I know who to contact if I have a healthcare concern | 31 |
| If I need to go to see a healthcare professional, I am able to get there | 34 |
| I usually bring someone along to help support me in my appointments | 31 |
| If I need to go to the pharmacy, I am able to get there | 32 |
| I am able to follow a procedure for next steps if asked (for example, I will attend follow-up appointments, annual checkups if applicable, etc...) | 33 |
| I am able to make appointments for myself | 32 |
| I will wait until it is an emergency before I go to see a healthcare professional | 31 |
| Chosen not to go in to see a healthcare professional regarding a health concern | 39 |
| ***System*** | |
| In most appointments, I have enough time to discuss my concerns with healthcare professionals | 42 |
| If I need to go to see a specialist for a healthcare concern, I am able to do so | 46 |
| I often choose not to go to the doctor with concerns if I need to see a specialist because I know that it will take me many appointments before I can see the specialist | 42 |
| I usually leave my appointments knowing what the next steps are (i.e. follow-up appointments, medications, etc. | 43 |
| I am provided with appropriate support after I receive a diagnosis of any kind (i.e. anything from infections to chronic conditions) | 43 |
| ***Sensory experiences*** | |
| Reported at least one sensory difference (hyper- or hyposensitivity) | 0 |
| I am able to describe how my symptoms feel in my body | 13 |
| I am able to describe how bad my pain feels | 12 |
| I am able to describe my sensory processing differences to healthcare professionals | 103 |
| The sensory environment of the waiting room is more overwhelming than other environments | 13 |
| The sensory environment of the office is more overwhelming than other environments | 15 |
| My senses frequently overwhelm me so that I have trouble focusing on conversations with healthcare professionals | 13 |
| ***Triggers for a shutdowns*** | |
| The idea of going to see a healthcare professional | 60 |
| Setting up an appointment to see a healthcare professional | 60 |
| Sensory environment of the waiting room | 61 |
| Sensory environment of the office | 66 |
| Seeing a different healthcare professional to  whom you expect | 61 |
| Talking to a healthcare professional | 60 |
| Picking up a prescription | 62 |
| Having to see many healthcare professionals before being able to talk to a specialist | 57 |
| After a diagnosis of any kind due to lack of follow-up or support | 70 |
| ***Triggers for a meltdown*** | |
| The idea of going to see a healthcare professional | 60 |
| Setting up an appointment to see a healthcare professional | 60 |
| Sensory environment of the waiting room | 61 |
| Sensory environment of the office | 66 |
| Seeing a different healthcare professional to  whom you expect | 61 |
| Talking to a healthcare professional | 60 |
| Picking up a prescription | 62 |
| Having to see many healthcare professionals  before being able to talk to a specialist | 57 |
| After a diagnosis of any kind due to lack of  follow-up or support | 70 |
| **Health outcome items** | |
| ***Rates of conditions overall*** | |
| Rates of diagnosed mental health conditions | 2 |
| Rates of diagnosed physical health conditions | 2 |
| Rates of suspected mental health conditions | 1 |
| Rates of suspected physical health conditions | 0 |
| Rates of healthcare professional recommended mental health assessments | 0 |
| Rates of healthcare professional recommended physical health assessments | 0 |
| ***Rates of individual diagnosed conditions*** | |
| Dementia | 0 |
| Arthritis | 0 |
| Blindness/partial sight | 0 |
| Breathing conditions | 0 |
| Cancer | 0 |
| Deafness of hearing loss | 0 |
| Diabetes | 0 |
| Heart conditions | 0 |
| High blood pressure | 0 |
| Intellectual disability | 0 |
| Kidney or liver disease | 0 |
| Neurological condition | 0 |
| Stroke | 0 |
| Anorexia nervosa | 3 |
| Anxiety | 0 |
| Attention deficit hyperactivity disorder | 0 |
| Binge eating | 1 |
| Bipolar disorder | 0 |
| Bulimia | 0 |
| Depression | 0 |
| Insomnia | 1 |
| Obsessive-compulsive disorder | 0 |
| Panic disorder | 0 |
| Personality disorder | 0 |
| Post-traumatic stress disorder | 0 |
| Postnatal depression | 0 |
| Schizophrenia | 0 |
| Seasonal affective disorder | 0 |
| Self harm | 0 |

**Table S3:** Missing participants per survey item for cisgender autistic and TGD autistic compared to cisgender non-autistic individuals.
.

| **Survey item** | **Number of participants with missing data** |
| --- | --- |
| ***General healthcare experiences*** | |
| Are you able to see healthcare professionals as often as you would like? | 63 |
| Do you have health insurance? | 5 |
| ***Communication*** | |
| I am usually able to explain what my symptoms are | 50 |
| I usually understand what my healthcare professional means when they discuss my health | 50 |
| I do not usually ask all the questions I would like to about my health | 57 |
| I can bring up a health concern even if my healthcare professional doesn’t ask about it | 56 |
| I know what is expected of me when I go to see my healthcare professional | 50 |
| ***Anxiety*** | |
| The idea of going to see a healthcare professional makes me feel anxious | 67 |
| The environment of the waiting room office makes me feel anxious | 70 |
| I feel anxious when I see a different healthcare professional to whom I expect | 71 |
| The process of setting up an appointment makes me anxious | 71 |
| The process of picking up a prescription makes me anxious | 76 |
| I frequently leave my healthcare professional’s office feeling as though I did not receive any help at all | 70 |
| ***Access and advocacy*** | |
| I know who to contact if I have a healthcare concern | 231 |
| If I need to go to see a healthcare professional, I am able to get there | 98 |
| I usually bring someone along to help support me in my appointments | 97 |
| If I need to go to the pharmacy, I am able to get there | 97 |
| I am able to follow a procedure for next steps if asked (for example, I will attend follow-up appointments, annual checkups if applicable, etc...) | 99 |
| I am able to make appointments for myself | 101 |
| I will wait until it is an emergency before I go to see a healthcare professional | 98 |
| Chosen not to go in to see a healthcare professional regarding a health concern | 95 |
| ***System*** |  |
| In most appointments, I have enough time to discuss my concerns with healthcare professionals | 137 |
| If I need to go to see a specialist for a healthcare concern, I am able to do so | 141 |
| I often choose not to go to the doctor with concerns if I need to see a specialist because I know that it will take me many appointments before I can see the specialist | 141 |
| I usually leave my appointments knowing what the next steps are (i.e. follow-up appointments, medications, etc. | 145 |
| I am provided with appropriate support after I receive a diagnosis of any kind (i.e. anything from infections to chronic conditions) | 144 |
| ***Sensory experiences*** |  |
| Reported at least one sensory difference (hyper- or hyposensitivity) | 0 |
| I am able to describe how my symptoms feel in my body | 31 |
| I am able to describe how bad my pain feels | 31 |
| I am able to describe my sensory processing differences to healthcare professionals | 856 |
| The sensory environment of the waiting room is more overwhelming than other environments | 37 |
| The sensory environment of the office is more overwhelming than other environments | 36 |
| My senses frequently overwhelm me so that I have trouble focusing on conversations with healthcare professionals | 33 |
| ***Triggers for a shutdowns*** |  |
| The idea of going to see a healthcare professional | 167 |
| Setting up an appointment to see a healthcare professional | 185 |
| Sensory environment of the waiting room | 180 |
| Sensory environment of the office | 186 |
| Seeing a different healthcare professional to  whom you expect | 181 |
| Talking to a healthcare professional | 180 |
| Picking up a prescription | 187 |
| Having to see many healthcare professionals  before being able to talk to a specialist | 176 |
| After a diagnosis of any kind due to lack of  follow-up or support | 195 |
| ***Triggers for a meltdown*** |  |
| The idea of going to see a healthcare professional | 167 |
| Setting up an appointment to see a healthcare professional | 185 |
| Sensory environment of the waiting room | 180 |
| Sensory environment of the office | 186 |
| Seeing a different healthcare professional to  whom you expect | 181 |
| Talking to a healthcare professional | 180 |
| Picking up a prescription | 187 |
| Having to see many healthcare professionals  before being able to talk to a specialist | 176 |
| After a diagnosis of any kind due to lack of  follow-up or support | 195 |
| **Health outcome items** | |
| ***Overall rates of conditions*** | |
| Rates of diagnosed mental health conditions | 0 |
| Rates of diagnosed physical health conditions | 0 |
| Rates suspected mental health conditions | 1 |
| Rates of suspected physical health conditions | 0 |
| Rates of healthcare professional recommended mental health assessments | 0 |
| Rates of healthcare professional recommended physical health assessments | 0 |
| ***Rates of individual diagnosed conditions*** | |
| Dementia | 0 |
| Arthritis | 0 |
| Blindness/partial sight | 0 |
| Breathing conditions | 0 |
| Cancer | 0 |
| Deafness of hearing loss | 0 |
| Diabetes | 0 |
| Heart conditions | 0 |
| High blood pressure | 0 |
| Intellectual disability | 0 |
| Kidney or liver disease | 0 |
| Neurological condition | 0 |
| Stroke | 0 |
| Anorexia nervosa | 1 |
| Anxiety | 0 |
| Attention deficit hyperactivity disorder | 0 |
| Binge eating | 0 |
| Bipolar disorder | 0 |
| Bulimia | 0 |
| Depression | 0 |
| Insomnia | 0 |
| Obsessive-compulsive disorder | 0 |
| Panic disorder | 0 |
| Personality disorder | 0 |
| Post-traumatic stress disorder | 0 |
| Postnatal depression | 0 |
| Schizophrenia | 0 |
| Seasonal affective disorder | 0 |
| Self-harm | 0 |
